# Multiple Microelectrode Recordings in STN‐DBS Surgery for Parkinson's Disease: A Randomized Study

**DOI:** 10.1002/mdc3.12621

**Published:** 2018-05-08

**Authors:** Silje Bjerknes, Mathias Toft, Ane E. Konglund, Uyen Pham, Trine Rygvold Waage, Lena Pedersen, Mona Skjelland, Ira Haraldsen, Stein Andersson, Espen Dietrichs, Inger Marie Skogseid

**Affiliations:** ^1^ Department of Neurology Oslo University Hospital Oslo Norway; ^2^ Institute of Clinical Medicine University of Oslo Oslo Norway; ^3^ Department of Neurosurgery Oslo University Hospital Oslo Norway; ^4^ Department of Neuropsychiatry and Psychosomatic Medicine Oslo University Hospital Oslo Norway; ^5^ Department of Psychology University of Oslo Oslo Norway

**Keywords:** PD, motor fluctuations, STN‐DBS, multiple microelectrode recordings

## Abstract

**Background:**

Subthalamic nucleus deep brain stimulation improves motor symptoms and fluctuations in advanced Parkinson's disease, but the degree of clinical improvement depends on accurate anatomical electrode placement. Methods used to localize the sensory‐motor part of the nucleus vary substantially. Using microelectrode recordings, at least three inserted microelectrodes are needed to obtain a three‐dimensional map. Therefore, multiple simultaneously inserted microelectrodes should provide better guidance than single sequential microelectrodes. We aimed to compare the use of multiple simultaneous versus single sequential microelectrode recordings on efficacy and safety of subthalamic nucleus stimulation.

**Methods:**

Sixty patients were included in this double‐blind, randomized study, 30 in each group. Primary outcome measures were the difference from baseline to 12 months in the MDS‐UPDRS motor score (part III) in the off‐medication state and quality of life using the Parkinson's Disease Questionnaire‐39 (PDQ‐39) scores.

**Results:**

The mean reduction of the MDS‐UPDRS III off score was 35 (SD 12) in the group investigated with multiple simultaneous microelectrodes compared to 26 (SD 10) in the single sequential microelectrode group (*p =* 0.004). The PDQ‐39 Summary Index did not differ between the groups, but the domain scores activities of daily living and bodily discomfort improved significantly more in the multiple microelectrodes group. The frequency of serious adverse events did not differ significantly.

**Conclusions:**

After 12 months of subthalamic nucleus stimulation, the multiple microelectrodes group had a significantly greater improvement both in MDS‐UPDRS III off score and in two PDQ‐39 domains. Our results may support the use of multiple simultaneous microelectrode recordings.

**Trial registration:**

http://ClinicalTrials.gov Identifier: NCT00855621 (first received March 3, 2009).

## Introduction

Subthalamic nucleus deep brain stimulation (STN‐DBS) is a well‐established therapy for Parkinson's disease (PD) patients with motor fluctuations.^1^, ^2^, ^3^ However, the degree of improvement depends on accurate electrode placement in the sensory‐motor part of the STN.^4^ Stimulation near the dorsolateral border of the nucleus is most effective,^5^, ^6^ whereas stimulation of other parts of STN or nearby structures may cause adverse effects.^7^, ^8^

Targeting may be based on stereotactic imaging, clinical assessment during test stimulation, and/or microelectrode recordings (MER). The use of MER has been considered valuable in expert hands, but vary across centers, and randomized studies comparing MER strategies on outcomes have been warranted.^9^ Some centers use one MER and adding more if needed,^4^, ^10^ the single sequential approach, usually using the length of STN signals as a criterion. However, there is no certain significant relationship between STN length and UPDRS improvement.^11^ Also, with this approach, it may be difficult to recognize too medially placed electrodes.^12^ Additional trajectories should be separated by at least 2mm, due to a risk of entering the same tract.^13^ Therefore, many centers use a simultaneous insertion of multiple microelectrodes^6^, ^14^ that can reduce these problems and has shown superiority in clinical outcomes in non‐randomized studies.^15^

No randomized trial comparing multiple simultaneous MER to single sequential MER guidance during STN‐DBS surgery for PD has been reported. Our objective was to perform a controlled randomized study, to assess the value of three‐dimensional mapping of the STN on the motor outcome and health‐related quality of life.

## Methods

### Trial Design and Participants

In a single‐center study, patients were randomized 1:1 to receive intraoperative investigation primarily with single MER (sMER group), or five MERs (mMER group) on each side. In the sMER group, the protocol allowed for introducing additional MERs sequentially if the first trajectory was deemed electrophysiologically or clinically unsatisfactory. In the mMER group, only anatomical limitations allowed a reduction of MERs.

The inclusion and exclusion criteria for the participants are shown in Table 1. The investigation included cognitive screening, using the Mattis Dementia Rating Scale and assessment of self‐reported emotional symptoms by the Hospital Anxiety and Depression Scale (HADS), which yields sub‐scores for anxiety and depression.

**Table 1:** Inclusion and Exclusion Criteria

Inclusion criteria:
• Parkinson's disease according to the UK Brain Bank criteria
• Age < 75 years
• Disease duration ≥ 5 years
• UPDRS motor score ≥ 20 points in the medication‐off state
• > 30% reduction of non‐tremor motor score in medication‐on state (range 0 to 108) or severe levodopa unresponsive tremor
• Marked motor fluctuations with or without troublesome dyskinesias, and/or severe tremor, and/or intolerable side effects of dopaminergic drugs
• Failure of best oral medical treatment to sufficiently control symptoms
• Mattis Dementia Rating Scale score >130 (maximum 144)
Exclusion criteria:
• Previous surgery for Parkinson's disease
• Marked axial motor symptoms unresponsive to levodopa
• Brain MRI showing marked atrophy or white matter changes
• Increased risk of bleeding
• Comorbidities with short life expectancy
• Other surgical contra‐indications
• Dementia
• Unstable or major psychiatric disorders (including psychosis, major depression or severe anxiety disorder)
• Insufficient understanding of the Norwegian language (preventing participation in the psychiatric and neuropsychological evaluations)

Abbreviations: UPDRS, Unified Parkinson's disease rating scale.

### Neurological and Neuropsychiatric Evaluations

Patients were investigated preoperatively and postoperatively at 3 and 12 months with the Movement Disorder Society revision of the Unified Parkinson's Disease Rating Scale (MDS‐UPDRS) to assess non‐motor experiences of daily living (Part I), motor experiences of daily living (Part II), motor examination (Part III), and the severity and impact of motor fluctuations (Part IV).^16^ The MDS‐UPDRS III (range 0 to 132) was scored after overnight withdrawal of dopaminergic drugs (medication‐off) and after a levodopa dose approximately 1.5 times the patient's morning dose (medication‐on). Postoperative evaluations were always performed in the stimulation‐on state. As five individual raters performed the MDS‐UPDRS III examinations, inter‐rater reliability was evaluated in 17 patients from scores performed by two separate raters (Pearson correlation coefficient r = 0.98, *p <* 0.001). The Hoehn & Yahr scale (0 to 5) was scored using the recommendations of the MDS task force.^17^ Health‐related quality of life was assessed using the Parkinson's Disease Questionnaire‐39 (PDQ‐39), covering the eight domains—mobility, activities of daily living (ADL), emotional well‐being, stigma, bodily discomfort, social support, cognition, and communication). A score for each domain is calculated, and the mean across the domains to yield the Summary index (SI).^18^, ^19^ Surgical complications, adverse events, and changes in medication and stimulation parameters were documented. Serious adverse events (SAE) were registered as defined by the U.S. Food and Drug Administration.

A neuropsychological assessment was performed before surgery and after 12 months. A battery of validated neuropsychological tests covering attention/working memory,^20^ executive functions,^21^ verbal and visual memory,^22^ visuospatial memory,^23^ and processing speed^24^ was used. Raw scores were transformed into standardized T‐scores based on available normative data provided by the test publisher. A composite global cognitive index score was calculated expressing the mean of individual test T‐scores. A complete psychiatric evaluation was performed preoperatively and at 3 and 12 months.

### Randomization and Blinding

Patients were randomly assigned in 1:1 ratio to the sMER or mMER groups in blocks of 4 or 6, by a computerized randomization generator handled by the Office of Clinical Research, an independent body at Oslo University Hospital. The surgeon obtained the result the day before surgery. Both the patients and the neurologists performing post‐operative assessments remained blinded.

### Surgical and Radiological Procedure

The patient was withdrawn from dopamine agonist therapy one‐week preceding surgery and from levodopa and COMT‐inhibitors from midnight before surgery.

For surgical planning, T1 and T2 weighted 1.5T MRI series (2 mm slices) were fused with a CT scan (1.25 mm slices) performed after mounting the stereotactic frame and localizer, using the Brainlab iPlan system. The STN target was determined by direct targeting, with guidance from classical coordinates. The entry point was placed to obtain a trajectory that avoided sulci and ventricles, preferably in gyrus F2 anterior to the coronal suture.

In both groups, STar™ microdrive with the Ben‐Gun configuration was used, performing MER at 0.5–1 mm steps from 10 mm above the planned target to the ventral border of the STN or identification of substantia nigra signals, and the STN identified by a typical neuronal firing pattern.

#### sMER Group

A minimum of one central MER was inserted. If typical STN signals were recorded, test stimulation was performed from the macro contact of the microtargeting electrode (FHC microTargeting, 44975L). A neurologist evaluated effects on motor symptoms and side effects, from 1.5 mA and up to 4.5‐5 mA (130 Hz, 60 microseconds). If the central trajectory gave unsatisfactory recordings or clinical response, or side effects at thresholds lower than 3.0 mA, an additional MER trajectory 2 mm anterior, posterior, medial, or lateral to the initial one was inserted. This procedure could be repeated with up to five sequentially introduced MERs. The permanent electrode (Lead model 3389, Medtronic) was inserted in the chosen trajectory and fixed to the skull using the Stimloc system (Medtronic).

#### mMER Group

Five microelectrodes were introduced simultaneously. The trajectory judged to be located in the dorsolateral part of the nucleus and yielding the longest depth of good STN signals was chosen for clinical test stimulation. Test stimulation was then done as described above for the sMER patients. The trajectory with the best therapeutic window was used for the permanent electrode. The neurostimulator (Kinetra or Activa PC, Medtronic) and connection cables were implanted under general anesthesia. A postoperative CT was performed the next day.

### Initiation and Modulation of Stimulation Parameters

Stimulation was initiated using pulse width 60 microseconds and frequency 130 Hz, monopolar configuration, and amplitudes individually modified for each patient.

### Primary and Secondary Endpoints

Primary endpoints were the mean score differences of individual patients from preoperative to one year of STN‐DBS, in the medication‐off MDS‐UPDRS III, and the PDQ‐39 scores.

The MDS‐UPDRS III medication‐off score was also incorporated into the so‐called quality index as a secondary endpoint.^1^, ^25^ The individual patient's stimulation‐induced MDS‐UPDRS III score reduction (medication‐off) from preoperative to one year is divided by the reduction obtained by the preoperative levodopa challenge. Thus it expresses how much of the potential improvement predicted by the levodopa‐response that has been achieved with stimulation (where quality index > 1 indicates larger improvement and, < 1 smaller than predicted).

Prespecified secondary endpoints also included the mean score differences in individual patients of MDS‐UPDRS I, II, and IV scores, and of levodopa equivalent daily dose (LEDD). All other outcomes were analyzed post hoc.

### Sample Size and Statistical Analysis

The primary null hypothesis was an outcome of no difference in the score changes of the MDS‐UPDRS III medication‐off or PDQ‐39 between both randomization groups. We estimated that a sample size of 60 patients (30 per group) would power the study at 80% with a 5% probability of a type I error. This design allows detection of a 20% difference between treatment groups concerning the primary outcome criteria assuming SD of 25%, allowing for an overall dropout rate of 10%.

Primary and secondary endpoints were studied using t‐tests comparing group means of individual patient's score differences from baseline to 12 months of stimulation. Paired sample t‐test was used to evaluate score differences within each group. For PDQ‐39, 7% of the items were missing (no pattern recognized), preventing the calculations of domain scores and SI. Therefore, we performed multiple imputations (20 imputations) of missing items for intention‐to‐treat analysis, according to the CONSORT guidelines.^26^ All statistics were performed using IBM SPSS Statistics version 22.

### Ethical Considerations

All participants gave written informed consent before inclusion. The study was approved by the Regional Committee for Medical and Health Research Ethics (REC South East, project no. 6.2009.46), and registered at http://ClinicalTrials.gov (Identifier NCT00855621, first received March 3, 2009). The study is reported according to the CONSORT guidelines.^26^

## Results

Between April 2009 and December 2013, 76 patients underwent STN‐DBS surgery at Oslo University Hospital. Sixty patients (15 women, 45 men) participated in the study, and primary endpoint analysis included 55 patients (sMER group: 29, mMER group: 26; Figure 1). Table 2 shows median and range of the baseline demographic and clinical data for each group.

**Table 2:** Baseline Characteristics of the Study Population

	Total	Single MER	Multiple MER
Gender (n (%))			
Male	45 (75)	20 (67)	25 (83)
Female	15 (25)	10 (33)	5 (17)
Age	62 (44 to 71)	62 (44 to 71)	63 (49 to 70)
Disease duration (years)	11.0 (4 to 23)	11.0 (4 to 23)	11.5 (4 to 17)
HAD *			
Anxiety	4 (0 to 12)	3 (0 to 11)	4 (0 to 12)
Depression	3 (0 to 19)	3 (0 to 19)	3 (0 to 10)
Mattis Dementia			
Rating Scale**	142 (131 to 144)	142 (131 to 144)	141 (134 to 144)
LEDD	1291 (428 to 2490)	1347 (874 to 2259)	1174 (428 to 2490)
MDS‐UPDRS I	10.5 (1 to 25)	10.5 (1 to 25)	10.5 (3 to 24)
MDS‐UPDRS II	16.0 (0 to 32)	15.5 (0 to 31)	16.5 (9 to 32)
MDS‐UPDRS III off	47 (23 to 78)	44 (28 to 66)	51 (23 to 78)
MDS‐UPDRS III on	13 (1 to 45)	13 (3 to 37)	13 (1 to 45)
MDS‐UPDRS IV	10.0 (0 to 16)	10.5 (1 to 15)	9.5 (0 to 16)
HY off (n (%))			
1	0	0	0
1.5	0	0	0
2	20 (33)	10 (33)	10 (33)
2.5	17 (28)	9 (30)	8 (27)
3	10 (17)	7 (23)	3 (10)
4	11 (18)	4 (13)	7 (23)
5	2 (3)	0	2 (7)
HY on			
1	3 (5)	1 (3)	2 (7)
1.5	3 (5)	1 (3)	2 (7)
2	37 (62)	18 (60)	19 (63)
2.5	15 (25)	9 (30)	6 (20)
3	2 (3)	1 (3)	1 (3)
4	0	0	0
5	0	0	0

Abbreviations: MER, microelectrode recording; HAD, hospital anxiety and depression scale; LEDD, levodopa‐equivalent daily dosage; MDS‐UPDRS, Movement Disorder Society – Unified Parkinson's disease rating scale; HY, Hoehn and Yahr.

Values are medians (min‐max). N = 60, except for HAD (*58, 29 in each group) and Mattis dementia rating scale (**50, 23 in the sMER and 27 in the mMER group).

**Figure 1:**
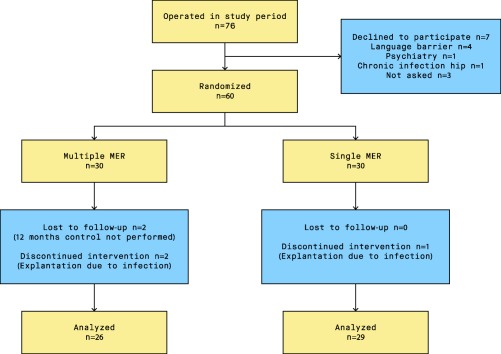
Participant flow chart. Abbreviations: MER, microelectrode recording.

Table 3 shows the distribution of MER trajectories per hemisphere in the two groups. In the sMER group, 50% were investigated with > 1 MER on at least one side. In the mMER group, 10% were investigated with < 5 MER. The central trajectory was chosen for the permanent electrode in 65% of sMER and 72% of mMER patients.

**Table 3:** Number of Patients with Different Combinations of Microelectrode Trajectories Recorded in the Two Randomization Groups

	Combinations of number of trajectories performed (left + right)	No of patients
sMER	1+1	15
	1+2	3
	2+1	2
	1+3	1
	3+1	1
	2+2	2
	2+3	1
	3+2	4
	3+3	1
mMER	5+5	27
	4+5	1
	5+4	1
	1+1	1

Abbreviations: sMER, single sequential microelectrode recording(s); mMER, multiple simultaneous microelectrode recordings.

For the whole population, the mean improvement from baseline to one year of individual patient MDS‐UPDRS III medication‐off scores was 30 (*p <* 0.001, 61%), and the mean quality index was 0.90. For PDQ‐39, significant improvements were found in the SI (*p <* 0.001) and the domains mobility, activities of daily living (ADL), stigma, cognition, and bodily discomfort (all with *p <* 0.023).

Mean stimulation parameters at one year were similar in both groups; sMER/mMER: 2.8/2.9 Volt, 137/134 Hz and 61/64 microseconds (all with *p >* 0.301). One of the two middle contacts was used in 96% of electrodes.

### Primary Endpoint Analyses

From baseline to one year, the mean improvement of individual patient MDS‐UPDRS III medication‐off scores was 35 in the mMER group and 26 in the sMER group (*p =* 0.004, Table 4).

**Table 4:** Change from Baseline to 12 Months of STN‐DBS for Primary and Secondary Endpoints

	Preoperative			12 months			Difference (effect size)			
	Total	Single	Multiple	Total	Single	Multiple	Total	Single	Multiple	p‐value
Primary endpoints										
n	60	30	30	55	29	26	55	29	26	
MDS‐UPDRS III Off	49 (13)	45 (10)	52 (15)	20 (9)	19 (9)	20 (9)	30 (11)	26 (10)	35 (12)	0.004
PDQ‐39 Summary index	26.7	25.4	28.0	21.1	22.8	19.1	5.2	2.9	7.8	0.100
Domains										
Mobility	34.2	32.4	35.9	27.2	30.4	23.6	6.5	2.2	11.3	0.110
ADL	36.1	32.9	39.4	22.8	24.6	20.8	13.6	8.3	19.6	0.024
Emotional	17.6	16.5	18.8	18.3	18.8	17.7	‐1.5	‐2.1	‐0.9	0.743
Stigma	25.4	24.0	26.8	15.9	17.5	14.2	8.5	5.8	11.6	0.204
Social Support	12.0	12.8	11.3	12.6	16.0	8.9	‐1.7	‐2.8	‐0.4	0.604
Cognition	24.2	25.4	23.0	19.8	20.0	19.6	4.5	6.1	2.8	0.397
Communication	19.5	16.1	23.0	25.6	24.5	26.8	‐6.2	‐7.9	‐4.3	0.543
Bodily Discomfort	44.6	43.4	45.7	26.3	31.0	21.0	17.8	13.1	23.0	0.048
Secondary endpoints										
Quality index							0.90 (0.29)	0.84 (0.25)	0.97 (0.32)	0.091
MDS‐UPDRS I	11.3 (6.0)	10.7 (5.8)	12.0 (6.4)	9.0 (5.7)	8.8 (5.5)	9.2 (5.9)	2.2 (4.5)	2.1 (4.6)	2.4 (4.5)	0.845
MDS‐UPDRS II	16.8 (7.3)	15.4 (8.1)	18.1 (6.2)	11.5 (6.7)	11.2 (6.8)	11.7 (6.7)	5.8 (7.2)	4.6 (6.8)	7.2 (7.5)	0.183
MDS‐UPDRS IV	9.6 (3.5)	10.0 (3.4)	9.3 (3.6)	2.6 (3.7)	2.9 (4.1)	2.4 (3.2)	7.2 (4.2)	7.1 (4.8)	7.3 (3.6)	0.886
LEDD	1301 (441)	1332 (332)	1269 (532)	639 (328)	652 (355)	624 (301)	655 (362)	686 (360)	620 (368)	0.503
MDS‐UPDRS III On*	14 (9)	13 (8)	16 (11)	12 (7)	11 (7)	13 (8)	4 (9)	3 (8)	5 (10)	0.401

Abbreviations: MDS‐UPDRS, Movement Disorder Society – Unified Parkinson's disease rating scale; PDQ‐39, Parkinson's disease questionnaire‐39; SI, summary index; ADL, activities of daily living; LEDD, levodopa‐equivalent daily dosage.

The differences (effect size) are the mean of individual patients score difference from baseline to 12 months of STN‐DBS. For PDQ‐39, SD is not presented as pooled output on multiple imputation data sets do not include this.

*Not a defined endpoint.

**Figure 2:**
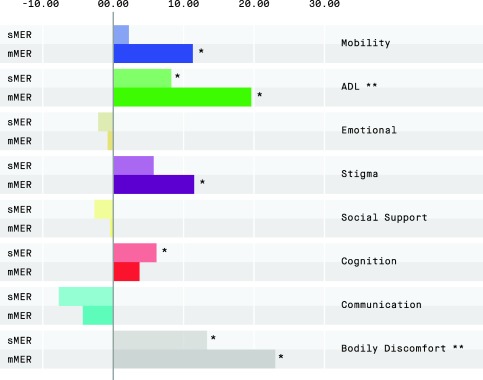
Mean change from preoperative to 12 months of STN‐DBS in the eight PDQ‐39 domains, comparing the two randomization groups. Abbreviations: PDQ‐39, Parkinson's disease questionnaire‐39; ADL, activities of daily living; sMER, single sequential microelectrode recording(s); mMER, multiple simultaneous microelectrode recordings. *p < 0.05 for change in each domain versus baseline. **p < 0.05 for between group differences.

Mean PDQ‐39 SI improvement was 7.8 for mMER (*p <* 0.001) and 2.9 for sMER (*p =* 0.208), but with no significant between‐group difference (*p =* 0.100, Table 4). Of the eight PDQ‐39 domains, four improved significantly in the mMER group versus three in the sMER group (Figure 2). ADL and bodily discomfort improved significantly more in the mMER group (*p =* 0.024 and *p =* 0.048).

### Secondary Endpoint Analyses

The mean quality index was 0.97 in the mMER group and 0.84 in the sMER group (*p =* 0.091). The total scores of MDS‐UPDRS I, II, and IV, and total LEDD, all improved significantly from baseline to 12 months in both randomization groups (*p <* 0.010), but not significantly different between the groups (Table 4).

### Post Hoc and Adjusted Analyses

Despite randomization, the mean preoperative MDS‐UPDRS III medication‐off score was higher in the mMER group than in the sMER group (not explained by preoperative tremor score differences; sMER mean 7[5] and mMER 8[7]). To adjust for this preoperative difference we performed a one‐way analysis of covariance (ANCOVA), with estimated means after adjustment for the preoperative MDS‐UPDRS III medication‐off difference being 29 for sMER and 32 for mMER (*p =* 0.24).

**Figure 3:**
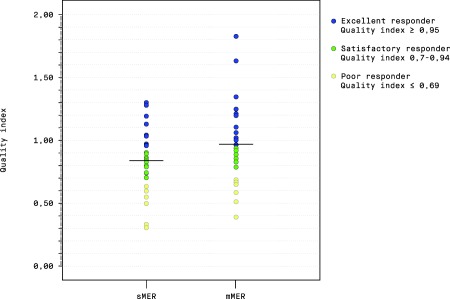
Responder analysis of the distribution of the quality index in the two randomization groups. Abbreviations: sMER, single sequential microelectrode recording(s); mMER, multiple simultaneous microelectrode recordings. In the mMER group, the proportion of excellent responders was 50% versus 35% in the sMER group, and the proportion of satisfactory responders 27% versus 45%. The proportions of poor responders were 23% and 21%, respectively.

A responder analysis based on the distribution of the quality index in the two randomization groups is shown in Figure 3.

We performed a post hoc analysis to compare the patients who ended up being evaluated with a total of 5 to 10 MER (n = 31), to those evaluated with only 2 to 4 MER (n = 24). The MDS‐UPDRS III medication‐off difference at 12 months was 34 and 26 (*p =* 0.004), and the quality index 0.97 and 0.81 (*p =* 0.039), respectively.

The global cognitive index was significantly reduced from baseline to follow‐up in both sMER and mMER groups (t = 3.01, *p =* 0.006 and t = 4.34, *p <* 0.001, respectively), but with no significant between‐group difference in magnitude of change (t = 1.82, *p >* 0.05).

### Adverse Events

There were no intracranial hemorrhages, epileptic seizures, or mortality. Serious adverse events (SAE) were registered in 31/60 patients. The incidence did not differ between the groups (*p =* 0.798, Table 5). Three patients (5%) had surgical site infections (SSI). In one sMER patient, infection over the extension cable spread to an intracerebral abscess, and in the mMER group, two had SSI in relation to the neurostimulator (*p =* 0.701). The only event registered as life‐threatening was the intracerebral abscess, which resolved after hardware removal and long‐term antibiotic treatment, and left no permanent sequelae. The only event registered as a permanent disability was a progressive cognitive deficit at the one‐year evaluation in a sMER patient.

**Table 5:** Serious Adverse Events (SAE)

	Total	sMER	mMER
Serious adverse events:			
Death	0	0	0
Life threatening	1	1	0
Permanent disability	1	1	0
Hospital admission	12	4	8
Intervention required	10	8	2
Other	7	2	5
**Total SAE**	**31**	**16**	**15**
Causes of Serious adverse events:			
**Infections**	**10**	**5**	**5**
‐ Surgical site infection	5	2	3
‐ Other infections	5	3	2
**Falls**	**10**	**6**	**4**
‐ without injury	2	2	0
‐ with fracture	5	3	2
‐ with brain concussion	2	0	2
‐ with scalp wound	1	1	0
**Neuropsychiatry**	**19**	**10**	**9**
‐ Hypomanic episode	5	3	2
‐ Depressive episode	5	3	2
‐ Anxiety	1	0	1
‐ Confusion	4	3	1
‐ Increased irritability	1	0	1
‐ Hallucinations/delusions	1	0	1
‐ Cognitive decline	2	1	1
**Other**	**2**	**0**	**2**
‐ TIA	1	0	1
‐ Hemorrhage (extracranial)	1	0	1

Abbreviations: sMER, single sequential microelectrode recording(s); mMER, multiple simultaneous microelectrode recordings.

In the sMER group four patients had 2 types of causes for SAE and one patient had two falls that both lead to fractures. In the mMER group three patients had 2 causes of SAE and one patient had 3 causes of SAE.

Other somatic SAE occurred in 12 patients (20%; *p =* 0.832), the most frequent being falls (17%). Neuropsychiatric SAE occurred in seventeen patients (28%), 9 in the sMER and 8 in the mMER group (*p =* 0.904). The most common neuropsychiatric SAE were hypomanic episodes, depressive episodes, and postoperative confusion.

## Discussion

This randomized trial is the first to evaluate the clinical impact of using multiple simultaneous MER versus single sequential MER to guide correct placement of permanent STN electrodes in PD patients. We found that the improvement in MDS‐UPDRS III medication‐off from baseline to 12 months was significantly larger in the mMER group. The PDQ‐39 domains ADL and Bodily discomfort also improved significantly more in the mMER group. The PDQ‐39 Summary Index improved significantly from baseline only in the mMER group.

The significantly larger difference in the MDS‐UPDRS III medication‐off scores found in the mMER group may partly be explained by the higher mean preoperative score in that group, despite randomization. These findings concur with a meta‐analysis, which found that higher UPDRS III at baseline predicted lower absolute UPDRS III scores postoperatively.^1^

The potential improvement of PD motor symptoms by STN‐stimulation can be predicted by the patient's improvement in the preoperative levodopa‐challenge.^13^, ^27^ How much of this potential improvement each patient has achieved, can be expressed by the quality index, first used by Benabid et al.^25^ This is an individualized quality marker of stimulation efficacy on motor symptoms, reducing the impact of interpersonal variability in preoperative disease severity. In our study the mean quality index points in the same direction as the crude MDS‐UPDRS III difference, being close to 1 in the mMER group (regarded as an optimal treatment effect), versus just above 0.8 in the sMER group. An excellent response (quality index ≥ 0.95; Figure 3) was achieved in 50 % of the mMER patients, versus 35% in the sMER group. The quality index is also useful when comparing results across different patient populations with variable severity of disease.^1^

As much as 50% of the sMER patients were evaluated with > 1 MER in at least one hemisphere, possibly leading to smaller differences in treatment efficacy between the two randomization groups. The permanent electrode was implanted in a different trajectory than the central (MRI based target) in 32% of hemispheres, with no significant group differences (sMER 35% versus mMER 28%). This figure is similar to other studies, reporting this in about one‐third of electrodes.^4^ One could speculate that the benefit of stimulation would be less if the central MRI based trajectory were chosen in all patients. Causes of deviations from planned central trajectory can be errors in the stereotactic procedure or brain‐shift. It has been reported that the majority of brain shift occurs before MER.^28^ One study reported that amount of brain shift impacted the number of MER trajectories needed to optimize targeting.^29^

Our patients perform well, also when compared with previous studies.^1^, ^25^ We underline that we have used the new MDS‐UPDRS, used in few of the previously published studies, but Goetz et al. showed that part III is comparable in both versions.^30^ The safety profile of our cohort is good, with no significant group differences in serious adverse events. Three patients (5%) had SSI, including one rare case of an intracranial abscess, which was the only case of intracranial infection observed in our 10‐year cohort of DBS treated patients.^31^ Also, no between‐group differences were found regarding neuropsychiatric adverse events or changes to global cognitive index.

One argument raised against using multiple simultaneous MER has been the risk for hemorrhage. The reported incidence of hemorrhage is varying between 1–20%.^32^, ^33^ Seijo et al. report decreasing frequency of hemorrhage,^33^ probably explained by raised awareness on avoiding the ventricles and sulci during lead insertion,^34^, ^35^ and improved surgical experience and expertise.^27^ Age and hypertension are also reported as risk factors for hemorrhage.^32^ No hemorrhages in 120 electrode placements indicate that our hemorrhage frequency is low. In our opinion, the clinical benefits of using multiple MER outweigh the relatively small risk of hemorrhage.

Technical advances in the field have emerged during recent years, where directional current steering and closed‐loop devices are promising.^36^, ^37^ However, these techniques also have limitations and their efficacy still depends on correct localization of the target. Neuroimaging techniques are advancing, but it is not certain that this will lead to more uniform targeting as experts may define the optimal STN target differently.^38^ One study showed that the final stimulation site, based on MER and intraoperative stimulation, was located more lateral and superior than both atlas and MRI based targets.^39^ Also, newer studies show discrepancies comparing borders on T2 and SWI MRI sequences compared to the electrophysiological STN.^40^, ^41^ Further research is needed to evaluate the impact of these newer techniques on target accuracy and clinical outcome.^42^

Even though MRI techniques have evolved over the last years, they will never be able to provide electrophysiological information about the target area. We consider that such information is beneficial for optimal target localization and that our findings indicate a benefit from using multiple MER introduced simultaneously during STN‐DBS‐surgery.

## Author Roles

1. Research Project: A. Conception, B. Organization, C. Execution; 2. Statistical Analysis: A. Design, B. Execution, C. Review and Critique; 3. Manuscript Preparation: A. Writing the First Draft, B. Review and Critique.

S.B.: 1B‐C, 2A‐C, 3A‐B

M.T.: 1A‐C, 2A‐C, 3A‐B

A.E.K.: 1C, 2B‐C, 3A‐B

U.P.: 1C, 2C, 3B

T.R.W.: 1C, 2B‐C, 3A‐B

L.P.: 1B‐C, 2C, 3B

M.S.: 1C, 2C, 3B

I.H.: 1B, 2C, 3B

S.A.: 1A‐B, 2B‐C, 3A‐B

E.D.: 1A‐B, 2C, 3B

I.M.S.: 1A‐C, 2A‐C, 3A‐B

Silje Bjerknes, Mathias Toft and Inger Marie Skogseid have had full access to all data, and take responsibility for the integrity of the data and the accuracy of the data analysis presented in the manuscript.

## Disclosures

**Ethical Compliance Statement**: We confirm that we have read the Journal's position on issues involved in ethical publication and affirm that this work is consistent with those guidelines. All participants gave written informed consent prior to inclusion.

**Funding Sources and Conflict of Interest**: This study has been supported by grants from the South‐Eastern Norway Regional Health Authority. It has not received any financial support from private corporations. Mathias Toft, Ane Konglund, Espen Dietrichs, and Inger Marie Skogseid have during the study period received speaking honoraria and travel support from Medtronic. Inger Marie Skogseid has also received honoraria for participating in international multi‐center studies of DBS in dystonia, supported by Medtronic. Silje Bjerknes and Mona Skjelland have received travel support from Medtronic. Lena Pedersen served as a fully employed PD nurse at the Department of Neurology, Oslo University Hospital during her work with the study. Starting from May 1, 2014 she was fully employed by Medtronic Norway, and has since then not been working with the study, except from critical review of the manuscript. The other authors have nothing to report.

**Financial disclosure for the previous 12 months**: In addition to the financial disclosures stated above, these contributors have received support from the following the past 12 months:

Mathias Toft, speaking honoraria from AbbVie, Roche, and Nordic Infocare. Espen Dietrichs, from AbbVie and Global Kinetics Corp. Inger Marie Skogseid from Medtronic and Desitin Pharma, for lectures at scientific meetings.
